# Effect of the Reciprocal Inhibition Technique on Pain, Range of Motion, and Functional Activities in a Patient With Upper Trapezitis: A Case Report

**DOI:** 10.7759/cureus.48127

**Published:** 2023-11-01

**Authors:** Swapna Jawade, Neha Chitale, Sakshi Padmawar, Pratik Phansopkar

**Affiliations:** 1 Musculoskeletal Physiotherapy, Ravi Nair Physiotherapy College, Datta Meghe Institute of Higher Education and Research (Deemed to be University), Wardha, IND; 2 Dr. D. Y. Patil College of Physiotherapy, Dr. D. Y. Patil Vidyapeeth Society, Pune, IND

**Keywords:** rehabilitation, physical therapy, pain, range of motion, reciprocal inhibition technique, muscle energy technique, trapezitis

## Abstract

In recent years, muscle energy techniques (METs) have become more widely accepted in conventional manual therapy as an alternate treatment for joint dysfunction. Neck pain is a common clinical condition that can occur in the presence or absence of an injury history, evidence of trauma, and/or favorable radiography results. A 32-year-old female with neck pain has reduced range, which leads to difficulty in her daily activities. The evaluation and treatment plan are described. The use of heat fermentation and reciprocal inhibition techniques with accurate time measurement has led to a reduction in pain and functional disability. We report that it created a carefully planned and extensive rehabilitation regimen for treating neck pain. This therapy helped therapists provide evidence-based care and learn new management techniques to include in their daily routines.

## Introduction

Muscle energy techniques (METs) have been used to correct cervical and thoracic range of motion (ROM) impairments for more than 40 years. Reciprocal inhibition is one of the manual techniques used for pain relief as well as to improve ROM [1]. The majority of MET studies that have been published have looked at how well METs affect ROM in the cervical and thoracic spine. Manual therapy is most frequently used to alleviate range-of-motion limits brought on by joint dysfunction in the cervical and thoracic spines [2]. In recent years, METs have become more widely accepted in conventional manual therapy as an alternate treatment for joint dysfunction. Although METs have been used in therapeutic settings, nothing is known about how they might affect ROM in those who use them [1]. The fourth most disabling musculoskeletal problem is neck pain, which is a frequent ailment. It is the 21st most severe ailment overall out of 291 conditions [2]. Neck pain is a common clinical condition that can happen whether there is previous trauma, evidence, or positive radiography results [3]. Evidence suggests that as people get older, neck pain will become more common [3]. There are numerous therapy options for mechanical neck pain. The best management, however, is still up for debate. Muscle imbalance can frequently be the secondary cause of pain, even if joint deterioration may occasionally be the initial cause [4]. Neck pain is a generalized pain with mechanical characteristics, such as symptoms brought on by holding certain neck positions, moving the neck, or palpating the cervical muscles [5]. Uncomfortable work postures, stress, anxiety, heavy lifting, and physically demanding jobs are common causes of neck pain [5]. According to the literature, neck muscular atrophy and weakening frequently cause neck pain and impairment [6]. Neck discomfort, accompanying disability, and cervicogenic headaches are all significantly negatively correlated with cervical muscular strength [7]. METs target both the active tone component as well as the passive component of the shortened muscle [8]. In terms of the pathophysiology of neck discomfort, articular issues or joint degeneration may be to blame, but more frequently, increased muscle tension and muscular imbalances are to blame, which can then produce an increase in joint reaction forces and poor posture. Stretching methods are therefore more effective at treating neck pain [9]. In contrast to typical stretching, METs are also advised in acute circumstances, along with muscle guarding and splinting [10].

## Case presentation

A 32-year-old female with right-hand dominance who is an employee in an IT company and has to work for nine hours a day was presented. She was alright one month ago when she experienced pain in the posterior aspect of her neck, and the pain aggravated on movements like lateral flexion and flexion of the neck. In August 2022, she visited an orthopedician in a local clinic, who prescribed her some pain-relieving medicines. She took them for five days, but the pain did not subside, and therefore she was referred to AVBRH for physiotherapy management. She came to the physiotherapy outpatient department in August 2022, where she was assessed for posture and neck movements and was diagnosed with right-side trapezitis. Post-diagnosis, physiotherapy management was initiated.

Clinical findings

On observing the patient, the body was mesomorphic. Her right shoulder was slightly elevated and adducted as compared to her left, and in lateral view, a forward head posture was seen. On palpation, a trapezius muscle spasm was found on the right side. Grade 2 tenderness was present. On the Numerical Pain Rating Scale (NPRS), she rated her pain as 7/10. The quality of the pain was dull-aching. The pain and stiffness increased in the morning.

Therapeutic intervention

**Figure 1:**
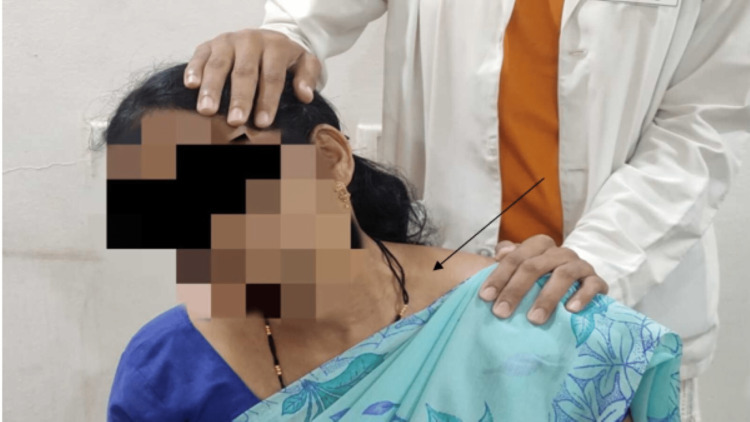
Shows trapezius stretching on left side.

For two weeks, the intervention program was followed five days a week (Table 1). Hot fermentation was given for 10 minutes, which was followed by a reciprocal inhibition technique for five repetitions, holding for five seconds each. In between, a rest period was given for five seconds for all the patients, and lastly, trapezius muscle stretching was performed for 30 seconds, three times. Figure 1 shows trapezius stretching for a 32-year-old female.

**Table 1:** Intervention protocol for a week.

Intervention	Duration
Hot fermentation	10 minutes
Reciprocal inhibition	5 repetitions × 5 seconds
Rest period	5 seconds
Trapezius stretching	30 seconds × 3 repetitions

Outcome measures

Outcome measures for pain, range, and functional activities were assessed on the first day and after two weeks by the Numerical Pain Rating Scale (Table 2), Range of Motion (Tables 3-4), and Neck Disability Index (NDI) - 8/50 and 3/50, respectively.

**Table 2:** Pre- and post-values of NPRS. NPRS: Numerical Pain Rating Scale.

NPRS	Baseline	After 2 weeks
On movement	7/10	3/10
On rest	2/10	1/10

**Table 3:** Baseline range of motion assessment. ROM: range of motion.

Movement	Active ROM (in degrees)	Passive ROM (in degrees)
Neck flexion	20	26
Neck extension	30	35
Lateral flexion (right)	15	18
Lateral flexion (left)	12	16
Rotation (right)	38	40
Rotation (left)	30	35

**Table 4:** Range of motion assessment after two weeks. ROM: range of motion.

Movement	Active ROM (in degrees)	Passive ROM (in degrees)
Neck flexion	35	38
Neck extension	40	45
Lateral flexion (right)	20	22
Lateral flexion (left)	19	21
Rotation (right)	42	45
Rotation (left)	40	42

## Discussion

Upper trapezitis has the potential to create neck pain, limit the cervical range of motion, and restrict functional activities and should therefore be addressed as part of a comprehensive physical therapy program [10]. Upper trapezitis can be treated with a wide variety of treatment protocols, with variable results. Owing to the heterogeneity of the existing trials, examining manual techniques suggests that such approaches may be effective; however, the extent of their effectiveness is currently unknown [10]. Pain is reduced by simultaneous isometric contraction and stretching, which excite mechanoreceptors and proprioceptors. In earlier investigations of the post-isometric relaxation technique of MET, where the above-mentioned outcome improved for diverse causes of neck pain, similar outcomes were achieved for pain reduction, improvement in cervical ROM, and functional activities [11]. By increasing stretch tolerance and changing the viscoelastic characteristics of the soft tissue, MET alleviates pain and promotes flexibility. Another study that examined the benefits of the post-isometric relaxation technique on mechanical neck pain came to the conclusion that MET greatly decreased pain and enhanced day-to-day functioning [5]. Similarly, a study found that MET is successful in reducing pain and increasing the range of motion [12-14]. There was a significant difference between the two groups, and a study found that both therapeutic strategies - muscular energy technique and static stretching - were effective in reducing mechanical neck pain. However, MET was superior to static stretching because it increased the active cervical range of motion while lessening pain severity [15,16].

## Conclusions

The most frequent cause of neck pain in adults is trapezitis, which is treatable with a precise rehabilitation plan and stops future deconditioning. In this example, there was a considerable decrease in discomfort, improvement in cervical range, and restoration of functional activities after two weeks of care. This case study creates a carefully planned and extensive rehabilitation regimen for treating neck pain caused by trapezitis. This therapy helps therapists provide evidence-based care and learn new management techniques to include in their daily routines. Additionally, this includes innovative advances in long-term management methods for neck pain.
